# Insights into the Current Trends in the Utilization of Bacteria for Microbially Induced Calcium Carbonate Precipitation

**DOI:** 10.3390/ma13214993

**Published:** 2020-11-05

**Authors:** Sing Chuong Chuo, Sarajul Fikri Mohamed, Siti Hamidah Mohd Setapar, Akil Ahmad, Mohammad Jawaid, Waseem A. Wani, Asim Ali Yaqoob, Mohamad Nasir Mohamad Ibrahim

**Affiliations:** 1 Centre of Lipids Engineering and Applied Research, Universiti Teknologi Malaysia, Skudai 81310 UTM, Johor, Malaysia; scchuo2@yahoo.com.my; 2 Department of Quantity Surveying, Faculty of Built Environment, Universiti Teknologi Malaysia, Skudai 81310 UTM, Johor, Malaysia; 3 Malaysia-Japan International Institute of Technology, Jalan Sultan Yahya Petra, Universiti Teknologi, Malaysia, Kuala Lumpur 54100, Malaysia; 4 Laboratory of Biocomposite Technology, Institute of Tropical Forestry and Forest Products (INTROP), Universiti Putra Malaysia, Serdang 43400 UPM, Selangor, Malaysia; 5 Department of Chemistry, Govt. Degree College Tral, Kashmir J&K-192123, India; waseemorg@gmail.com; 6 School of Chemical Sciences, Universiti Sains Malaysia, Penang 11800, Malaysia; asimchem4@gmail.com (A.A.Y.); mnm@usm.my (M.N.M.I.)

**Keywords:** bacteria, biocementation, construction, microbially induced calcium carbonate precipitation

## Abstract

Nowadays, microbially induced calcium carbonate precipitation (MICP) has received great attention for its potential in construction and geotechnical applications. This technique has been used in biocementation of sand, consolidation of soil, production of self-healing concrete or mortar, and removal of heavy metal ions from water. The products of MICP often have enhanced strength, durability, and self-healing ability. Utilization of the MICP technique can also increase sustainability, especially in the construction industry where a huge portion of the materials used is not sustainable. The presence of bacteria is essential for MICP to occur. Bacteria promote the conversion of suitable compounds into carbonate ions, change the microenvironment to favor precipitation of calcium carbonate, and act as precipitation sites for calcium carbonate crystals. Many bacteria have been discovered and tested for MICP potential. This paper reviews the bacteria used for MICP in some of the most recent studies. Bacteria that can cause MICP include ureolytic bacteria, non-ureolytic bacteria, cyanobacteria, nitrate reducing bacteria, and sulfate reducing bacteria. The most studied bacterium for MICP over the years is *Sporosarcina pasteurii*. Other bacteria from Bacillus species are also frequently investigated. Several factors that affect MICP performance are bacterial strain, bacterial concentration, nutrient concentration, calcium source concentration, addition of other substances, and methods to distribute bacteria. Several suggestions for future studies such as CO_2_ sequestration through MICP, cost reduction by using plant or animal wastes as media, and genetic modification of bacteria to enhance MICP have been put forward.

## 1. Introduction

**Figure 1:**
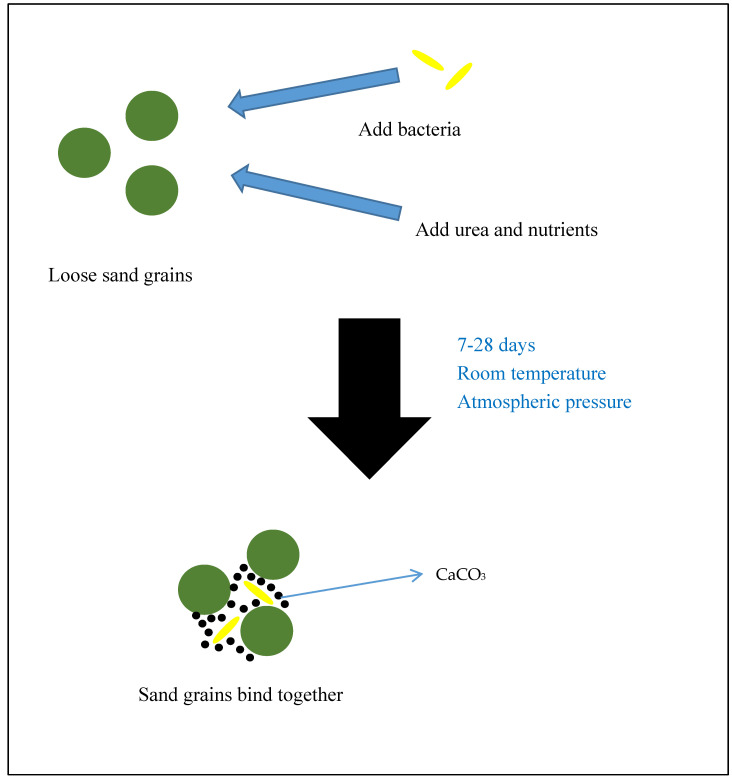
Schematic diagram of the microbially induced calcium carbonate precipitation (MICP) process for biocementation of sand.

Microbially induced calcium carbonate precipitation (MICP) is a process that occurs when microorganisms, especially bacteria, are provided with appropriate substrates and thus induce the formation of calcium carbonate (CaCO_3_) crystals. The CaCO_3_ formed is very useful in coating surfaces and binding different particles together [1,2,3]. MICP can occur under atmospheric pressure and other mild conditions. In fact, it happens in nature all around the world. This process has coated the surfaces of various natural structures and left hints about past ages for researchers to discover. Formation of CaCO_3_ by microorganisms has been studied through biomimetic approach and then applied in various fields such as construction, environment, geo-techniques, and nanotechnology [4,5]. An example of the MICP process is shown in Figure 1.

In recent years, there are increasing interests in MICP among researchers worldwide. A search in SciFinder with the keywords ‘MICP’ and ‘biocement’ showed increasing number of studies about MICP from the year 2010 to 2020. The increasing interest of researchers towards MICP and biocement may be due to increasing awareness on sustainability globally. A lot of studies focus on finding or developing sustainable materials and processes to replace conventional non-sustainable ones. The construction industry is one of the fastest growing fields due to rapid urbanization [6]. Large amounts of building materials are being consumed every day to build, maintain, and renew various structures. These building materials, especially ordinary Portland cement (OPC) and concrete, are not sustainable. Construction consumes a lot of natural resources and energy, while at the same time contributing 50% of CO_2_ emission worldwide [7,8]. Therefore, it is desirable to change current building materials into sustainable ones. In addition, biocementation through MICP can be used to reduce usage of OPC.

**Figure 2:**
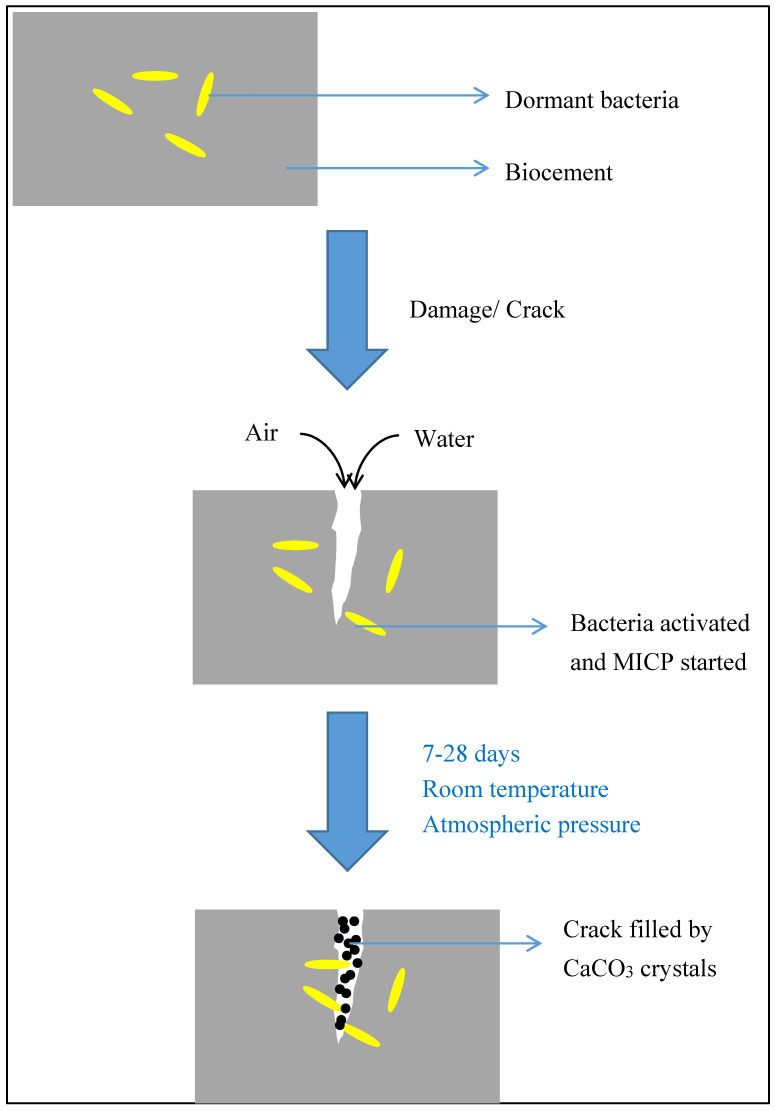
Schematic diagram for self-healing of biocement.

Construction costs are rising over the years and are expected to stay high for future times ahead. Producing building materials that are more durable and longer lasting can help to reduce maintenance costs. MICP was reported by various researchers to enhance strength and durability of building materials. Development of self-healing building materials also helps to reduce resources spent on routine repairs [9,10]. By including appropriate bacteria into cement or concrete, the formation of cracks will be stopped and sealed by the bacteria due to CaCO_3_ precipitation. Figure 2 shows the schematic diagram of self-healing process. Bacterial solution can also be applied from outside to seal a cracked surface on old building materials.

Conducting an MICP process requires knowledge from different fields including biotechnology, geotechniques, civil engineering, material engineering, and nanotechnology [11]. Current trends of technology integration encouraged researchers to investigate applications of MICP for various purposes. Most recent studies on MICP focus on consolidation of sand and soil, self-healing concrete and crack sealing [12,13], and removal of heavy metals/ions from water [14,15]. Technology integration in construction and other industries will make the applications of MICP easier.

The presence of bacteria is crucial for MICP to occur. The bacteria produce necessary enzymes such as urease and carbonic anhydrase to convert appropriate compounds into carbonate ions [16]. These activities change the microenvironment to favor precipitation of CaCO_3_ in the presence of calcium ions. Surface charges of bacterial cells attract calcium ions and then the cells serve as precipitation sites for CaCO_3_ crystals. Some bacteria also produce extracellular polymeric substances (EPS) that can enhance the MICP process. Different bacteria have been studied for their MICP potential. The bacteria must have high cell availability and high enzyme activity because they are often placed in harsh environments associated with high alkalinity, lack of nutrients, and high compressive force [17]. Bacterial strain and medium composition will affect CaCO_3_ crystal morphologies (calcite, vaterite and aragonite); thus, affecting the stability and strength of structures formed [18]. Therefore, careful considerations are required when choosing bacteria and its medium to obtain desired products.

The purpose of this paper was to review the bacteria used in some of the most recent studies of MICP. By learning and comparing the behavior of the bacteria and the results of MICP processes, insights on choosing suitable bacteria for certain applications have been proposed. This paper will thus help in future studies to further improve the MICP processes.

## 2. Bacteria Used in MICP

Globally, different bacterial strains have been tried by different researchers for MICP. Some of the commonly used bacterial strains that were successfully employed for MICP are discussed in the succeeding sections.

### 2.1. Sporosarcina pasteurii

*Sporosarcina pasteurii*, previously known as *Bacillus pasteurii* is the most commonly used bacterium for studying MICP due to its high urease activity. It is a non-pathogenic bacterial strain. Urease catalyzes the hydrolysis of urea to form ammonia and carbonic acid as shown in Equations (1) and (2).

CO(NH_2_)_2_ + H_2_O ⟶ NH_2_COOH + NH_3_ (1)

NH_2_COOH + H_2_O ⟶ NH_3_ + H_2_CO_3_ (2)

Ammonia then forms ammonium and hydroxide ions in water (Equation (3)).

NH_3_ + H_2_O ⟷ NH_4_^+^ + OH^−^ (3)

Carbonic acid also forms bicarbonate and hydrogen ions in water (Equation (4)).

H_2_CO_3_ ⟷ HCO_3_^−^ + H^+^ (4)

Formation of hydroxide ions causes pH to increase and shifts the bicarbonate equilibrium. This causes the formation of carbonate ions. The overall equation becomes as shown below (Equation (5)).

HCO_3_^−^ + H^+^ + 2NH_4_^+^ + 2OH^−^ ⟷ CO_3_^2−^ + 2NH_4_^+^ + 2H_2_O (5)

In the presence of calcium ions, calcium carbonate crystals can be precipitated as shown in Equation (6).

Ca^2+^ + CO_3_^2−^ ⟷ CaCO_3_ (6)

Table 1 shows some studies on sand and soil improvements using MICP with *Sporosarcina pasteurii*.

In laboratory scale experiments, researchers often test the biocementation potential of MICP by *Sporosarcina pasteurii* on sand columns composed of various types of sands and additives. Strength and durability of the sands are often enhanced after the MICP process. The research group of Cardoso et al. [19] used *Sporosarcina pasteurii* for biocementation of sand columns and showed that compressibility and tensile strength increased while permeability was decreased after MICP. They also showed that addition of clay in the sand column further enhanced its properties. Bu et al. [20] studied biocementation of sand using *Sporosarcina pasteurii* and compared with sands treated with normal cement or lime. Sands treated with MICP had higher unconfined compressive strength (UCS) than sands with 10% cement and flexure strength similar to sands with 20–25% cement. Lime treated sands were the weakest among those samples. Porter et al. [21] investigated the combinations of MICP by *Sporosarcina pasteurii*, OPC, and metakaolin to treat sand columns. Combination of *Sporosarcina pasteurii* and OPC had better performance in terms of UCS and water absorption as compared to other combinations or single treatment. Analysis revealed that MICP enhanced bridges formed by OPC or metakaolin between sand particles. A total of 93% of CaCO_3_ crystals formed at the bridging zones in sand columns treated by *Sporosarcina* *pasteurii* and OPC. This study suggested that there is synergistic relationship between chemical and microbial cementation process. Choi et al. [22] used *Sporosarcina pasteurii* for biocementation of PVA fiber reinforced sand columns. They found that at fixed CaCO_3_ concentration, increasing PVA fiber content further increased UCS and splitting tensile strength and decreased permeability of the sand columns. They also found that brittleness of sand column was greatly reduced with addition of fiber. They suggested that addition of fiber enhanced biocementation by filling more pores. Xiao et al. [23] showed that MICP by *Sporosarcina pasteurii* enhanced cyclic shear resistance of calcareous sand. The authors found that increasing biocementation solution can further reduce degree of sand deformation and increase liquefaction resistance due to more and bigger CaCO_3_ crystals filling voids. The CaCO_3_ crystals made the surface rougher and that enhanced bonding between sand particles. Sasaki and Kuwano [24] used *Sporosarcina pasteurii* to consolidate sands with different non-plastic fines content. They found that presence of fines content greatly reduced liquefaction resistance after MICP due to smaller void ratio, formation of smaller and unevenly distributed CaCO_3_. Higher concentration of biocementation solution or more cycles of treatment were suggested to enhance sand liquefaction resistance. Salifu et al. [25] treated sloped sand with MICP by *Sporosarcina pasteurii* and then tested it with tidal cycles. Sand slope angle was maintained after 30 simulated tidal cycles. This indicated that MICP process successfully stabilized and reduced erosion of slope surface significantly.

A critical analysis of these research reports indicates that *Sporosarcina pasteurii* has been effectively used by different research groups for compressibility and tensile strength enhancement, biocementation, UCS, and water absorption. The utilization of *Sporosarcina pasteurii* has been cost effective too, in addition to being effective.

There are several studies that have discussed the factors affecting MICP in sand columns. Tang et al. [26] stated that CaCO_3_ content from MICP in sand columns is affected by flow rate and hold time of biocementation solution. CaCO_3_ content in sand columns decreased at high flow rate and its distribution depended on hold time. They reported that sand columns treated with MICP by *Sporosarcina pasteurii* can achieve compressive strength of 3.29 MPa at 3 h hold time and 0.5 mol/L biocementation solution. Omoregie et al. [27] used different strains of *Sporosarcina pasteurii* to treat sand columns. They optimized the temperature, initial pH, incubation time, and urea concentration for MICP process. Their results indicated that final enhancement varied according to the strains of bacteria used. Duo et al. [28] used *Sporosarcina pasteurii* for biocementation of desert aeolian sand. They studied the effects of urea-CaCl_2_ concentration on CaCO_3_ amount, dry density, permeability, and UCS of the sand columns after MICP. All properties increased with higher urea-CaCl_2_ concentration, which showed that the formation of CaCO_3_ crystals consolidated the sand column. USC of sand columns was greatly increased when more than 14% CaCO_3_ formed. Their study showed that CaCO_3_ crystals mostly formed at sand particle surfaces and pores between sand particle when the concentration of urea-CaCl_2_ used was 0.5–1.0 mol/L. Solidifying and connecting properties became more significant at 1.5–2.5 mol/L urea-CaCl_2_. Methods to apply bacteria and cementation solutions into sand columns affect the MICP performance. Similar study was also carried out by Sharaky et al. and applied biocementation solution including *Sporosarcina pasteurii* for consolidation of sand columns [29]. Minto et al. [30] used *Sporosarcina pasteurii* for MICP on marble grains in columns and found that only the top to middle portion of the columns were solid enough. Rate of MICP increased while porosity and permeability decreased towards top of columns. This is due to the formation of CaCO_3_ during MICP that blocked the path and affected flow pattern of biocementation solution. Rowshanbakht et al. [31] used *Sporosarcina pasteurii* to enhance sand columns through a two phase-injection method. Bacteria retention, optical density (OD), and urease activity were optimized at 2/3 injection pore volume and 85% sand relative density. Maximum UCS was achieved at 1/3 injection pore volume and 85% sand relative density. The author found that the portion of the sand column near the injection point had a lower UCS than the other end. Permeability was found to decrease with increasing injection pore volume and sand relative density. More calcite formed near the injection point and calcite content throughout the sand column varied from 4.5% to 8%. Kakelar et al. [32] replaced yeast extract using sodium acetate in ratio for MICP of *Sporosarcina pasteurii* and concluded that cost can be reduced by using this technique. Minto et al. [33] applied MICP on sandstone cores through continuous injection of *Sporosarcina pasteurii* and nutrients. After that, they tested the sample with acidic fluids. Less than 1% permeability drop was reported due to CaCO_3_ blocking preferential flow paths and also buffered the acidic fluids. Tobler et al. [34] studied the transportation of *Sporosarcina pasteurii* in sandstone. The bacteria cells were found easily trapped in sandstone. Higher injection rate can enhance cells transportation. Although initial injection can distribute the cells uniformly, they were easily trapped on CaCO_3_ after MICP started. The authors stated that transport behavior must be determined for each bacteria strain individually.

A critical analysis this section indicates that injection method is superior to mixing method for the consolidation of sand columns using *Sporosarcina pasteurii*. Various factors (injection rate, injection pore volume, medium, etc.) need to be considered while designing an MICP process using *Sporosarcina pasteurii.*

Consolidation of soils through MICP is also frequently investigated. Grabiec et al. [35] mixed *Sporosarcina pasteurii* in silty soils to make cylinder samples. They found that soil shear strength and rigidity increased while soil deformation under stress reduced after the MICP process. This method further ensured soil lithification. The authors also demonstrated that high mechanical pressure involved during sample making may reduce bacterial survival rate, although their study showed that a number of compaction strokes had insignificant effect on bacterial survival in soil. Canakci et al. [36] used *Sporosarcina pasteurii* to consolidate organic soil and found that MICP was able to improve soil shear strength due to enhanced cohesion and internal friction. However, it was limited by strength of organic particles in the soil. Around 20% CaCO_3_ formed after MICP process. This is less than the amount of CaCO_3_ formed in sandy soil from other studies and therefore organic matter may have inhibited growth of CaCO_3_ crystals. Feng et al. [37] used simulation to study biocementation of soil through MICP by *Sporosarcina pasteurii* and OPC. They claimed that mechanical properties of bio-cemented sand can be predicted through careful calibration.

In addition to consolidation of sands and soils, researchers also showed great interest in making bacterial-based bricks, concretes, and mortars [38]. Some works are shown in Table 2. Bernardi et al. [39] made bio-bricks with silica rich masonry sand through MICP by using *Sporosarcina pasteurii*. They recorded the transition of bio-bricks from ductile to brittle within 28 days curing time. Up to 2.2 MPa compressive strength was achieved for their bio-bricks. Cuzman et al. [40] made bio-blocks with sand though MICP by *Sporosarcina pasteurii* with cement kiln dust as the calcium source. Ground granulated blast furnace slug was also added as a mean for solid waste recycling. Addition of solid wastes was shown to reduce urease activity due to high alkalinity and inhibitory effects. Nevertheless, this study suggested that it is a possible method to reduce construction costs and environmental pollution. There are many factors that need to be considered when making bacteria-based materials. Okyay and Frigi Rodrigues [41] attempted to optimize MICP by *Sporosarcina pasteurii* through a center composite design by varying the concentrations of urea, CaCl_2_, and nickel nitrate. Concentrations of urea and CaCl_2_ were identified as the significant factors. High urea to CaCl_2_ ratio enhanced the MICP process. No clear relation between bacterial growth and rate of MICP was observed. Zhang et al. [42] studied the effects of calcium source on MICP by *Sporosarcina pasteurii* in mortar samples. The calcium sources tested were calcium acetate, calcium chloride, and calcium nitrate. The amount of CaCO_3_ formed and water adsorption of sand column was not affected by type of calcium sources tested. However, samples using calcium acetate had the highest UCS and tensile strength. Those samples also had smaller and more uniformly distributed pore structure. This is related to the formation of 88% aragonite and 12% calcite in samples using calcium acetate, while only calcite formed in samples using other two calcium salts.

Many studies were conducted to enhance MICP by *Sporosarcina pasteurii* so that better materials can be produced at lower costs. Amiri and Bundur [43] compared the effects of different nutrients and calcium salts on MICP by *Sporosarcina pasteurii* to make mortar samples. Similar bacterial growth was observed in corn steep liquor (CSL) and yeast extract, but cells in CSL had lower surface charge. Both nutrients caused the setting time to increase but CSL caused it less than yeast extract. CSL also produced more CaCO_3_ than yeast extract after 28 days. However, compressive strength of yeast extract sample was higher than CSL sample. The authors also stated that calcium salts affect CaCO_3_ crystal morphology due to their different solubility.

Since the cost of the nutrient source can be up to 60% of total costs, it is obvious that the use of cheaper alternatives can reduce the cost of biocement production. In this direction, the research group of Yoosathaporn et al. [44] used chicken manure effluent as an alternative nutrient source for *Sporosarcina pasteurii* to make biocement cubes. The biocement had 30.27% higher compressive strength, 5.38% higher density, 3.2% more voids, and slightly higher water adsorption than normal cement. It was also documented that calcite and vaterite were formed. The authors reported that chicken manure effluent enabled more than two times urease production than commonly used nutrient broth, and was 88.2% cheaper. Critically speaking, it is pertinent to mention that chicken manure effluent–urea medium may create tiny air bubbles within cement that can weaken the cement structure and therefore more studies should be conducted to find low cost nutrient sources for biocement production.

Harsh conditions in cement and concrete often lead to low viability of bacterial cells. Williams et al. [45] simulated the harsh conditions in concrete and studied the effects on *Sporosarcina pasteurii*. Cell viability was greatly decreased at high temperature and high alkaline conditions. Urease activity was halted in high alkaline condition and greatly reduced at temperature higher than 45 °C. However, urease activity was not solely affected by cell viability. In order to ensure sufficient cells for MICP, carriers can be used to protect the cells from direct contact with its surroundings. This technique is often used by researchers to make self-healing samples. Amiri et al. [46] studied the effects of encapsulating *Sporosarcina pasteurii* in Air Entraining Agents (AEA) surfactant in cement mortar. They found that AEA has insignificant effects on cells zeta potential and in-vitro MICP but cell viability was greatly reduced. MICP process in mortar did not differ much with the addition of AEA. The author stated that AEA may encapsulate bacteria and the surfactant tails prevented water and nutrient from reaching the bacteria, thus killing them. This encapsulation method should be tested on bacteria endospores.

Looking at the research reports discussed above, it is very important to consider the effects of the process conditions (temperature, alkaline and acidic conditions, and carriers such as surfactants) on the urease activity of *Sporosarcina pasteurii*.

MICP can be applied on various surfaces by immersion or spraying method. Usually, the purpose of the processes is to heal cracks on the surface and to increase durability of the material. Choi et al. [47] studied the potential of *Sporosarcina pasteurii* bacterial solution to treat cracks on mortar samples. The authors applied treatment cycle once per day and found that seven cycles sealed most small cracks less than 0.52 mm and 21 cycles seal all cracks up to 1.64 mm. A portion of water permeability was recovered and only 8% tensile strength was recovered. This is due to the fact that all the voids in the cracks were not filled by CaCO_3_ crystals, and therefore adhesion and bridging effects were weak. Analysis revealed 1–2 mm CaCO_3_ layer at crack surface with a mix of calcite and vaterite. Balam et al. [48] compared MICP by *Sporosarcina pasteurii* and *Bacillus subtilis* to reduce water adsorption of various types of concrete aggregates categorized by their weight. They found that *Sporosarcina pasteurii* is better than *Bacillus subtilis* in reducing water adsorption of aggregates by up to 20%. The percentage of water adsorption reduction that was achieved varied from 0.6% to 28.2% depending on type of aggregates due to different microstructure and pore distribution. Bacteria concentration also affected the result. Generally, water adsorption reduction by MICP was more effective on lightweight aggregates compared to normal weight aggregates. Nosouhian et al. [49] showed that MICP by *Sporosarcina pasteurii* for surface treatment of concrete can help to enhance durability of concrete exposed to sulphate condition.

Some structures have very different environments such as those at subsurface. Verba et al. [50] used *Sporosarcina pasteurii* to make biocement-sandstone. The experiment environment was adjusted to mimic subsurface conditions with brine, 10 MPa high pressure, and supercritical CO_2_. Bacteria growth was greatly reduced by the presence of brine, although MICP still occurred in this condition. High pressure (10 MPa) and CO_2_ concentration did not have significant effects on *Sporosarcina pasteurii*. However, temperature at 40 °C greatly reduced the bacterial density. Calcite, vaterite, and aragonite were observed after MICP process. The result from this study is beneficial for subsurface MICP applications such as wellbore sealing. Cunningham et al. [51] also conducted similar studies on sandstone core for mitigation of wellbore leakage. MICP by *Sporosarcina pasteurii* greatly reduced sample pore size at 75.8 bar. It also greatly reduced permeability with the presence of brine but delayed sealing was observed. Usage of excess chemicals was suggested for field applications. Cunningham et al. [51] suggested using less expensive nutrient sources to reduce cost from $2.34 per liter to $0.28 per liter. Their field application was successful to reduce permeability and enhance wellbore integrity of a well in Alabama within four days. Phillips et al. [52] conducted a field scale study on wellbore cement sealing using MICP by *Sporosarcina pasteurii*. They reported that after four days of treatment, injectivity and pressure falloff were greatly reduced while solid content was greatly increased.

During formation of CaCO_3_ crystals, some other metal ions can also bind together and onto the crystals. This phenomenon has been exploited to remove metal contaminants. Mugwar and Harbottle [14] tested the potential *Sporosarcina pasteurii* to remove various heavy metals through MICP. They reported the following findings: complete removal of up to 0.5 mM Zinc in 7 days; near complete removal of up to 1.5 mM cadmium in 3 days; almost complete removal of up to 5 mM lead in 1 day; and almost complete removal of up to 0.01 mM copper in 1 day. The author stated that removal of heavy metals may be due to sorption or co-precipitation of the metals on or within CaCO_3_ crystals during MICP process.

Carbonates other than CaCO_3_ can also be precipitated by using appropriate sources. Yu et al. [53] used *Sporosarcina pasteurii* to treat loose quartz sand in columns through injection method. Magnesium chloride (MgCl_2_) was used instead of calcium salts; thus, magnesium carbonates were precipitated. The number of injections varied from 2 to 6 injections. They found that hydraulic conductivity, porosity, and maximum defect volume decreased with the number of injections. On the other hand, compressive strength and density increased with number of injections. They also showed that the application of biocementation solution through spraying method only once was sufficient to reduce wind erosion rate of the sand column to zero. Ruan et al. [54] used *Sporosarcina pasteurii* isolated from activated sludge to treat cracks in reactive magnesia cement. Magnesium carbonate was formed on crack surfaces and completely healed cracks wider than 0.15 mm after two cycles of treatment. They noted that urea concentration did not improve the healing process but affected pH and carbonate morphology.

Overall, *Sporosarcina pasteurii* has been extensively explored for the induction of MICP in different kinds of structures in different conditions. Several reports demonstrated the urease producing ability of this bacterial strain that bestows it with the effectivity of inducing MICP in different media and different environments. The optimization of the process conditions forms the corner stone of MICP processes using *Sporosarcina pasteurii.*

### 2.2. Bacillus sphaericus

*Bacillus sphaericus*, now reclassified as *Lysinibacillus sphaericus*, is an aerobic Gram-positive, mesophilic, rod shaped bacterium commonly found in soil and aquatic habitats. This bacterium is often used to produce mosquitocide [65,66]. It is able to produce urease and is tolerant to high alkalinity. Therefore, it is also often used in MICP experiments. Table 3 shows some of the works conducted. Moravej et al. [67] used *Bacillus sphaericus* for biocementation of dispersive soil. They optimized the MICP process at 1.5 bacteria OD, 7.5 g/L CaCl_2_ concentration, and 28 °C. The authors observed that calcite was formed that connected the soil grains together. Four to five days of treatment was enough to consolidate the soil. The effect of soil pH during treatment was also studied and it was observed to decrease greatly until day four to neutral. The pH change reduced double layer thickness and stabilized exchangeable sodium ions; thus, reducing dispersity in soil. Gupta et al. [68] added *Bacillus sphaericus* immobilized in biochar to make mortar samples. Biochar as carrier protect and distribute bacteria more uniformly throughout the mortar samples. They also added superabsorbent polymer to provide moisture for bacteria in mortar and polypropylene microfiber to reduce crack width and promote better self-healing. Addition of superabsorbent polymer decreased the compressive and flexural strength of mortar. These mortar samples showed more than 90% crack sealing for all crack width observed with 77% reduced water penetration and near 100% strength regained. Analysis showed that calcite was formed from the MICP process. Seifan et al. [69] reported that aeration with sufficient oxygen enhanced MICP of the *Bacillus sphaericus* and *Bacillus licheniformis*. The bacteria were able to tolerate pH up to 12. Higher pH enhanced MICP and caused smaller crystals to form. Their study showed that more vaterite was formed at pH 9–10 while more calcite was formed at pH 11–12. The same research group [70] also optimized MICP of *Bacillus sphaericus* and *Bacillus licheniformis* by adding oxygen releasing compounds. They suggested that the addition of oxygen releasing compounds in self-healing concrete healed cracks deep inside the concrete. In another investigation, the same group [71] investigated the effect of cell immobilization of *Bacillus sphaericus* and *Bacillus licheniformis* on magnetic iron oxide nanoparticles. It was found that when magnetic iron oxide nanoparticles exceeded 150 µg/mL, bacterial growth decreased but CaCO_3_ precipitation increased. The nanoparticles were absorbed on CaCO_3_ surface but did not affect crystal morphology. Thus, it was obvious that the use of various additives such as the nanoparticles may be employed to enhance concrete properties. It was further suggested that more studies are needed to fully investigate the interactions of bacteria with MICP for the production of materials with good strength, durability and self-healing ability. Shirakawa et al. [72] treated fiber reinforced cement with *Bacillus sphaericus* and then left the cement for 22 months exposed outdoors. They reported that MICP treatment with live *Bacillus sphaericus* with calcium acetate, yeast extract, glucose, and urea gave the best biodeterioration resistance, low water absorption, and porosity. The MICP process formed the smallest calcite crystals and more homogenous layer on cement surface to provide better protection.

### 2.3. Bacillus megaterium

*Bacillus megaterium* is a Gram-positive, rod shaped bacterium. It is the largest of all Bacillus species [77]. It was used as a model organism for various research before *Bacillus subtilis* was introduced. It is also ureolytic, thus being studied for MICP potential. Dhami et al. [78] used *Bacillus megaterium* solution to treat sand columns of different grain sizes from 0.2 mm to 1.5 mm. Cell viability was reported to be lower in bigger grain size samples initially, but the cell viability difference among all grain sizes became smaller later as MICP proceeded. The study showed that initial MICP for smaller grain size samples was higher but rate of increase over time was slower, while the opposite trend was observed for bigger grain size samples. Analysis showed that mostly calcite was formed from the MICP process. The authors also proposed an indirect method to measure the rate of MICP for site applications by effluent chemical analysis.

Although some recent studies showed the potential of *Bacillus megaterium* in making bacteria-based materials, most other studies are focused on its MICP behaviors. Jiang et al. [79] studied the ureolytic activity of *Bacillus megaterium* in oxic and anoxic conditions. They found that anoxic conditions enhanced the ureolytic activity, thus claiming that the bacteria have potential use in sub-seafloor environment with low temperature, high pressure, and anoxic condition. Bains et al. [80] investigated the effects of EPS produced by *Bacillus megaterium* SS3 on its MICP. They found that culture media affected bacteria growth, enzyme production, and EPS production. Calcium consumption was greatly increased at higher EPS concentration because EPS provided more nucleation sites for MICP. Dhami et al. [81] compared the effects of urease and carbonic anhydrase produced by *Bacillus megaterium* SS3 on MICP. During the MICP process, urease was found to maintain alkaline pH. Carbonic anhydrase showed a better MICP rate than urease during the initial 4 h while urease had a better MICP rate afterwards. The best pH and temperature reported for urease were 9 and 35 °C, respectively, while for carbonic anhydrase the values were 8 and 40 °C, respectively. Urease inhibitor caused greater MICP reduction compared to carbonic anhydrase inhibitor indicating that urease may be the main CaCO_3_ producer. The study showed that urease and carbonic anhydrase work synergistically for the MICP process. The authors in their other study [82] attempted to optimize media for *Bacillus megaterium* SS3 to obtain best MICP performance. They found that glucose and peptone are the best carbon and nitrogen sources for the bacteria. They also found that glucose, urea, and NaHCO_3_ had significant positive effects on the MICP performance. The optimized media was able to increase CaCO_3_ production by 70%. Table 4 shows some of the works conducted.

Overall, *Bacillus megaterium* has been successful in treating sand columns of different grain sizes. Bacterial concrete has been developed by using *Bacillus megaterium* in combination with recycled aggregates and nanosilica. The bacterium has affected the formation of materials in different environments effectively. However, further research is needed to optimize the parameters for MICP using *Bacillus megaterium*.

### 2.4. Bacillus subtilis

*Bacillus subtilis* is an aerobic, Gram-positive, spore forming, and rod-shaped bacterium commonly found in soil, water, and plants. It can produce various metabolites and has great potential in industrial applications [85,86,87,88]. It is non-ureolytic but studies have shown that that it has functional urease and can be activated though specific procedures. Although the exact mechanism is unknown, it is able to induce CaCO_3_ precipitation in appropriate media in the presence of calcium. It can also survive in harsh conditions, and thus has been studied for the preparation of bacteria-based materials. Mondal and Ghosh [89] added *Bacillus subtilis* at 103, 105, or 107 cell/mL cell densities to make mortar samples. The highest compressive strength increase was obtained at 105 cell/mL bacteria and the lowest water adsorption was obtained at 107 cell/mL bacteria. Self-healing potential of the mortars was shown to increase with higher bacteria density. Cracks up to 1.2 mm wide were completely healed within 28 days with 107 cell/mL bacteria. The author stated that higher bacteria concentration caused more CaCO_3_ crystals to form at surface thus providing better protection and reduce water permeation into inner layer. This condition may have affected cement hydration process and caused lower compressive strength at 107 cell/mL compared to 105 cell/mL bacteria. Mortar properties can be controlled by bacteria type, water to cement ratio, and cement to sand ratio. Perito et al. [90] found that a solution with dead *Bacillus subtilis* cells was able to precipitate CaCO_3_. The advantage is that dead cells have high heat resistance (up to 100 °C). In addition, calcite is produced only on the dead cell wall; thus, the MICP process can potentially be controlled. The authors used it to treat stones and an Angera Church wall. Water adsorption reduction of 16.7% on laboratory stones and 6.8% on Angera Church after treatment was reported. In addition to CaCO_3_, *Bacillus subtilis* was reported to produce phosphates when suitable chemicals were added and biosandstone with compressive strength of 2.1 MPa was obtained [91]. Table 5 shows some of the works conducted.

The reports discussed above indicate that *Bacillus subtilis* is activated through specific procedures. Its utilization in MICP processes ensures the formation of concrete with appreciable compressive strength. The MICP processes can also be controlled by tuning certain parameters.

### 2.5. Bacillus mucilaginous

Little information about *Bacillus mucilaginous* is available. It is known to produce carbonic anhydrase [94]. Carbonic anhydrase is able to extract CO_2_ from air or glucose for CaCO_3_ precipitation. Dhami et al. [82] compared the performance of bacteria urease and carbonic anhydrase for MICP. Urease has better MICP than carbonic anhydrase. Carbonic anhydrase absorbed CO_2_ from air, but the CaCO_3_ produced was less than what was obtained by using NaHCO_3_. Range of CaCO_3_ crystals formed among all samples was 151–189 mg/mL. The authors also reported that CaCO_3_ morphology was affected by bacterial species and carbon sources. It was also documented that urease mainly produced calcite while carbonic anhydrase produced vaterite. Qian et al. [95] used *Bacillus mucilaginous* to seal cracks on mortar samples and reduce their efflorescence. Surface treatment of mortar was not effective for reducing water adsorption. However, immobilization with agar layer greatly reduced the water adsorption to 14% of control sample. The authors demonstrated that agar significantly strengthened the bonding of deposit layer with mortar surface to form a dense film. The bacterial treatment also reduced mortar surface efflorescence by 42.4%. The advantage of using carbonic anhydrase producing bacteria is that they take CO_2_ from air and change it to HCO_3_^−^, which reacts with Ca(OH)_2_ to form CaCO_3_. Chen et al. [96] used ceramsite as carriers to encapsulate Bacillus mucilaginous and nutrients for preparation of biocement. Then the biocement was cracked and self-healing was observed. Cracks up to 0.5 mm wide were healed. The biocement has 0.8 × 10^−7^ m/s water permeability and flexural strength 3.3 times higher than normal cracked cement after 28 days of healing. Analysis showed that calcite was formed during the self-healing process. The study showed that immobilizing bacteria and nutrients with ceramsite can greatly enhance MICP process by increasing the amount of CaCO_3_ formed. Wang et al. [97] added *Bacillus mucilaginous* to make steel slag bricks and found that MICP greatly enhanced the bio-bricks. Up to 16.8 MPa compressive strength and 4.2 MPa flexural strength was recorded after three hours of MICP. Pore volume of bio-bricks was also greatly reduced after MICP. Analysis showed that MICP was detected up to 40 mm depth and CaCO_3_ was denser at surface because it is hard for CO_2_ to diffuse into the inner layer. The author suggested using higher pressure and more CO_2_ to enhance MICP in the inner layer of bio-bricks.

A critical analysis of this section indicates that *Baccilus mucilaginous* is a promising candidate for the production of self-healing biocement through MICP. The beauty of using *Bacillus mucilaginous* is that plenty of air surrounding the surfaces can be used a source of CO_2_, which then acts as the precursor for CaCO_3_ formation and deposition. However, further studies are warranted to ascertain the essentiality of this bacterium for MICP.

### 2.6. Cyanobacteria

Cyanobacteria are effective in sequestering atmospheric CO_2_. They can be modified to convert CO_2_ into valuable products through photosynthesis [98]. Therefore, they have the potential for MICP. Cyanobacteria have been reported to form intracellular CaCO_3_ [99]. These bacteria take calcium into cells and reduce the amount of calcium in solution, thus inhibiting CaCO_3_ formation in solution. Zhu et al. [100] studied the MICP of several cyanobacteria such as *Synechocystis* sp. and *Synechococcus* sp. on mortar surfaces. The cyanobacteria were either alive or killed by UV, and the MICP condition was either under light or in dark. All these conditions and bacterial species used affected the concentration of calcium consumed and sizes of CaCO_3_ crystals formed. The authors stated that the detailed mechanism to different calcification behaviors among cyanobacteria was not clear. Light intensity or UV exposure conditions may affect MICP depending on cyanobacterial species. The study showed that overall performance of *Synechocystis* sp. was better than *Synechococcus* sp. Zhu et al. [101] also studied the MICP of live and UV killed Gloeocapsa PCC73106 under light and dark conditions to treat mortar samples. The best properties among all samples studied were recorded for mortar treated with UV killed Gloeocapsa PCC73106, which had 7.7% higher compressive strength and 10% lower water adsorption compared to untreated mortar. The authors reported that more EPS was produced by UV-killed cells, and EPS protected bacterial cells from calcification, thus promoting cell adherence to mortar surface and also CaCO_3_ precipitation. Zhu et al. [102] studied the MICP of Synechococcus PCC8806 in cement mixture and on the concrete surface. In cement mixture, the bacteria with CaCl_2_ greatly enhanced CaCO_3_ precipitation in terms of size, amount, precipitation time, and CaCO_3_ morphology. Silicification occurred at cell surface before CaCO_3_ formation and may enhanced rate of CaCO_3_ precipitation. On the concrete surface, the bacteria produced 200 µm–270 µm thick CaCO_3_ layer. Water adsorption of the concrete was 3 g/cm^2^ after surface treatment. The CaCO_3_ layer was resistant to scratching with 4% mass lost after sonication test. Bundeleva et al. [103] studied the MICP behavior of *Gloeocapsa* sp. f-6gl. They found that light is important for its MICP process because there was no biomass increase in dark condition. There was no clear relation between biomass and rate of MICP. Only calcite was detected in almost all samples.

A critical analysis of this section indicates that cyanobacteria are potential microorganisms for MICP. The main merits of using cyanobacteria are that they do not need urea and a carbon source as they simply they take CO_2_ from atmosphere. Additionally, they do not produce nitrogen-based byproducts, and the costs of the processes are lower.

### 2.7. Other Bacteria

Several other bacterial strains have been potentially used for inducing MICP under various conditions in the laboratory setups. *Bacillus cereus* is an ureolytic, aerobic, Gram-positive, and rod-shaped bacterium commonly found in soil and food. Some strains of *Bacillus cereus* are harmful to humans, thus careful selection must be made to ensure safety [104]. Li et al. [105] tried MICP by *Bacillus cereus* NS4 to make mortar with addition of metakaolin. The bio-sample has higher compressive strength and lower permeability than normal mortar. Addition of 25% mass metakaolin gave better compressive strength and permeability compared to 0% or 50% mass metakaolin. Rozenbaum et al. [106] treated tuffeau stone with MICP by *Bacillus cereus* and then investigated the water transfer behavior in the stone through modeling. The authors documented that bio-coating has a limited lifetime, thus needing renewal within some period. Zhu et al. [107] reported that *Bacillus cereus* can be used for large scale nickel removal from soil. Their study showed that nickel concentration was reduced from 400 mg/kg soil to 38 mg/kg soil. Most nickel was bound to carbonate after treatment. The study showed that MICP by *Bacillus cereus* can be a potential alternative for large remediation of metal contaminated soil. Zhang et al. [108] added non-ureolytic and alkaliphilic *Bacillus cohnii* encapsulated in expanded perlite or expanded clay as healing agents into concrete mixture and evaluated self-healing capability. This bacterium utilizes organic compounds such as calcium lactate instead of urea to induce CaCO_3_ precipitation. The experiment showed that maximum crack width healed within 28 days was 0.79 mm for expanded perlite sample, 0.45 mm for expanded clay sample, 0.39 mm for bacterial samples without carriers, and 0.25 mm for normal concrete. Calcite was detected in all bacterial concretes. Expanded perlite had several advantages over expanded clay as carrier. It has high porosity and water adsorption to contain 12% more bacteria than expanded clay. Volume of expanded perlite used was 89% of expanded clay to contain same amount of *Bacillus cohnii*. The structure of expanded perlite allows it to provide more oxygen and water for MICP process. It is also protected by geopolymer coating. Furthermore, it costs only USD 0.22 per kg. The study suggests that expanded perlite can be good carrier for bacteria in making self-healing materials. Lors et al. [109] used *Bacillus pseudofirmus* solution with calcium lactate as calcium salt, calcium nitrate as inorganic salt, and yeast extract as nutrient to heal autogenously healed mortar that had been left for one year. Addition of calcium nitrate enhanced bacterial growth but did not improve CaCO_3_ precipitation. Nevertheless, it was able to enhance healing slightly. Calcite was detected from the MICP process. The authors stated that organic calcium salt should have organic part that can be used as nutrient for bacteria while anion of inorganic calcium salts need to be in some part of reaction so that CaCO_3_ crystals can form. Sharma et al. [110] prepared spores of *Bacillus pseudofirmus* DSM 8715. The spores were used to make mortar and treat cracks on concrete through injection method. This bacteria strain has great spore forming and germination properties but suitable germinants such as alanine, inosine, or NaCl are needed. MICP of this bacterium produced calcite and aragonite. Results showed that cracks were healed and water absorption of concrete was restored to normal value. Helmi et al. [111] studied the MICP of ureolytic bacterium, *Bacillus licheniformis*. They found that media having calcium as a pure source enhanced MICP but calcium acetate inhibited MICP due to pH decrease. Optimum pH was 8 and optimum temperature was 35 °C. Analysis showed that 89% calcite and 11% vaterite were formed. Bhaskar et al. [63] used *Sporosarcina ureae* encapsulated in zeolite to make bacterial mortars. The prepared mortars had better properties than normal mortars, but still not as good as mortars made using *Sporosarcina pasteurii*. Zhan et al. [112] reported that *Paenibacillus mucilaginosus* can absorb CO_2_ from air for MICP to bind fugitive dust. Erşan et al. [113] studied the potential of two nitrate reducing bacteria *Pseudomonas aeruginosa* and *Diaphorobacter nitroreducens* to make self-healing mortars with expanded clay or granular activated carbon as carriers. Their results showed that cracks up to 400 µm wide can be healed within 28 days using bacteria in expanded clay and cracks up to 500 µm wide can be healed within 56 days using bacteria in granular activated carbon. Up to 85% water tightness regain was reported for using bacteria in granular activated carbon. Calcite and aragonite were observed from the healing process. This study showed that nitrate reducing bacteria can have similar MICP performance as ureolytic and aerobic bacteria. Bai et al. [114] presented visual observations of MICP in *Pseudomonas aeruginosa* biofilm. They reported different MICP behavior compared to other studies. Lin et al. [115] studied the crystal morphology of CaCO_3_ formed by sulfate reducing bacterium, *Desulfovibrio bizertensis*. They found that CaCO_3_ crystal morphology is determined during the nucleation stage. They also found that the presence of phosphate inhibited the formation of aragonite.

Overall, a large diversity of bacteria have been investigated for MICP. Exciting results have been reported in terms of compressive strength of the materials developed and the overall efficiency of the MICP processes. However, more investigations are needed to explore the potential of these bacterial strains as far as MICP is concerned.

### 2.8. Bacteria Isolated from Various Environments

Researchers have been diligently searching for new bacterial species with the hope of achieving more efficient MICP. Diverse classes of bacteria have been isolated from various environments and tested for their MICP potential. Li et al. [116] isolated urease producing bacteria namely *Acinetobacter* sp. SC4 from Yixing Shanjuan Cave, China and tested its MICP potential to repair cracked masonry cement mortar. The repaired cement mortar regained 97.7% of its original compressive strength and has 42.4% lower water adsorption compared to repaired normal mortar. Analysis showed that calcite was formed through the MICP process. Zhang et al. [117] isolated a *Bacillus* sp. strain H4 from a mangrove conservation area in Shenzhen Bay, China and then developed a self-healing system using the bacteria together with oxygen releasing tablets. The oxygen releasing tablets were made from various peroxides and organic acids. Addition of oxygen releasing tablets was shown to greatly enhance the MICP process. Another study [118] also reported that high concentrations of a nitrate and calcium source inhibited the MICP process. Surrounding pH must be controlled at 9.5–11 because this bacteria strain cannot tolerate high alkalinity. Achal and Pan [119] used *Bacillus* sp. CR2 isolated from mine tailing soil of Urumqi, Xinjiang, China and studied the effects of calcium source on its MICP process. The calcium sources tested were CaCl_2_, calcium oxide, calcium acetate, and calcium nitrate. Results showed that CaCl_2_ was the best for enhancing bacteria growth profile, urease activity, and CaCO_3_ precipitation. Lv et al. [120] studied the stability of vaterite formed by *Lysinibacillus* sp. GW-2 isolated from soil in Nanjing Botanical Garden, China. They observed the formation and transitions of different CaCO_3_ crystals. They reported that organic matters allowed vaterite to remain stable without transforming to other morphology. *Lysinibacillus* sp. YS11 isolated by Lee et al. [121] is able to form spores, EPS, and biofilms. The bacteria showed MICP only in aerobic conditions with sufficient aeration. Xu et al. [122] tested the MICP potential of Microbacterium sp. GM-1 isolated from active sludge. They reported that urea concentration was the most significant factor for the MICP and calcite was the dominant crystals formed. Javadi et al. [123] made bio-blocks with recycled concrete aggregates and natural aggregates through MICP by urease producing bacteria *Staphylococcus pasteurii* isolated from a soil sample. There was less than 10% difference between UCS of bio-blocks made with those aggregates, and the maximum UCS obtained was 10 MPa. UCS of the bio-blocks decreased at higher temperature due to calcination and thermal decomposition of CaCO_3_ crystals. UCS also decreased after several freeze-thaw cycles with recycled concrete aggregates of bio-blocks having greater lowering due to higher water absorption. UCS could be reduced by increasing CaCO_3_ content to lower water absorption and ensure the distribution of tensile force in the bio-blocks. Vashisht et al. [124] isolated *Lysinibacillus* sp. from alluvial soil and sewage samples collected from different locations of district Solan, India and then made self-healing concrete with the bacteria. The self-healing concrete had 34.6% higher compressive strength than normal concrete. The authors claimed that their concrete had better self-healing ability than concrete made with *Bacillus megaterium*. Siddique et al. [125] isolated ureolytic bacteria *Bacillus aerius* strain AKKR5 from marble sludge to make bacterial concrete with cement baghouse filter dust replacing up to 30% of ordinary Portland cement. Bacteria concrete without cement baghouse filter dust had 10% higher compressive strength than normal concrete. However, addition of cement baghouse filter dust reduced the overall concrete properties shown by decreased compressive strength, increased water absorption, chloride permeability, and porosity. Analysis revealed that calcite and ettringite were formed from the process. In another study, the same group [126] also used the *Bacillus aerius* strain AKKR5 to make bacterial concrete with rice husk ash replacing up to 20% of ordinary Portland cement. Best bacterial concrete properties were obtained using 10% rice husk ash with 14.7% higher compressive strength than normal concrete, 0.8% water absorption, 1.5% porosity, and very low to moderate chloride permeability. Analysis showed that mainly calcite was formed from the process. Krishnapriya et al. [127] isolated some alkali resistant urease producing bacteria viz. *Bacillus megaterium*, *Bacillus licheniformis*, and *Bacillus flexus* from cement factory soil at Coimbatore, Tamil Nadu, India and used them to make bacterial concrete. They all enhanced concrete compressive strength and self-healing capability but not as good as commercial *Bacillus megaterium* MTCC 1684. The authors stated that enhancement of concrete properties is related to the ability of the isolates to form endospores. The *Bacillus flexus* isolate had limited endospore form, thus its bacterial concrete had lower performance compared to the other isolates. Hao et al. [128] used a *Bacillus* sp. strain isolated from soil sample from Perth, Australia for MICP surface treatment of polypropylene before making fiber reinforced cementitious composites. Compressive strength of the composites was decreased by 6.9% but energy adsorption capacity increased by 69.3%. Surface treatment of polypropylene enhanced the bending strength of the composites especially after cracking occurred. The author found that slight deposition of (0.026 g) was too weak to enhance the composites while heavy deposition of CaCO_3_ (0.372 g) made the CaCO_3_ layer too brittle and easily de-bonded from polypropylene. Thus, moderate deposition of CaCO_3_ (0.094 g) was suggested for surface treatment of polypropylene. Montano-Salazar et al. [129] isolated some bacteria from buildings in the National University of Colombia and tested their MICP potential. Nine isolates showed MICP potential but CaCO_3_ crystal morphology obtained was different between bacteria strains. *Psychrobacillus psychrodurans* M414 was identified as the best CaCO_3_ producer and was used to make bacterial mortar. The study showed that concrete compressive strength was greatly increased through immersion in biocementation solution but only slight increase of compressive strength was observed for direct addition of bacteria into concrete mixture. Mwandira et al. [130] isolated ureolytic bacteria *Pararhodobacter* sp. from soil near beachrock in Okinawa, Japan and then used the bacteria to treat lead contaminated sand columns. The contaminant was completely removed through co-precipitation with calcite or vaterite. Maximum UCS obtained was 1.33 MPa for fine sand sample, 2.87 MPa for coarse sand sample, and 2.80 MPa for mixed sand samples. Erşan et al. [131] isolated *Pseudomonas aeruginosa* and *Diaphorobacter nitroreducens* from soil and tested their MICP potential through denitrification in a minimal nutrient condition. The CaCO_3_ precipitation recorded was 53–72% of using optimal growth conditions. The author claimed that those bacteria have potential use for soil enhancement due to high CaCO_3_ precipitation in anoxic and minimum nutrient conditions. The bacteria also have potential use in self-healing concrete because they are concrete compatible and no other additives are needed. The research group of Daskalakis et al. [132] isolated *Bacillus pumilus* from a cave in Paiania, Athens, Greece and tested its potential for vaterite precipitation on stone marbles. Temperature and medium concentration were identified as the significant factors. Stone surface was completely covered by vaterite within 9 days and the vaterite was stable even after 1 year. The authors documented that acetate enhanced vaterite formation while the bacteria maintained vaterite stability. Charpe et al. [133] prepared bacteria solution from soil samples collected from Visvesvaraya National Institute of Technology campus in India without isolating the bacteria and then added the solution into cement mixture to make biocement. The biocement had 47.96 MPa compressive strength and 5.8% water adsorption. Analysis revealed that calcite and aragonite were formed during the process. The author claimed that biocement production costs can be reduced by using soil without isolating the bacteria, using lentil seed powder as the protein source, and sugar as the carbon source. Liu et al. [134] reported that some desert soil bacteria were able to utilize atmospheric CO_2_ for MICP. The MICP capability depends on the bacteria species.

A large number of bacterial strains have been isolated from diverse sources with varied conditions. The isolated and collected strains of bacteria have been investigated for MICP processes under different conditions. The bacteria have been successfully utilized in the formation of biocement via the precipitation of CaCO_3_ crystals. Of course, different methodologies for the precipitation of CaCO_3_ utilizing the bacterial strains isolated from diverse sources have been developed. Additionally, different MICP parameters have been optimized for enhancing the efficiency of the developed processes. However, further studies are needed to fully optimize the process conditions for the enhancement of MICP efficiency in terms of cost and applicability.

### 2.9. Unidentified or Unknown Bacteria

Some reports did not reveal the exact species of the bacteria used. Nevertheless, these reports can show some MICP behaviors and applications as references for future studies. Seifan et al. [135] studied the effects of several variables on MICP of various bacteria. They identified that bacteria species, concentration of bacteria, yeast extract, CaCl_2_, urea, and agitation speed were the significant factors. High bacteria concentration enhanced MICP. Too much yeast extract (more than 3 g/L) greatly reduced MICP. CaCl_2_ was said to be better than calcium lactate, calcium nitrate, or calcium acetate. Too low or high Ca^2+^ concentration will decrease MICP; thus, it must be controlled carefully. On the other hand, temperature was reported to have insignificant effect on MICP. CaCO_3_ crystal morphologies observed were only calcite and vaterite. More calcite was formed when using calcium lactate while more vaterite was formed using CaCl_2_. High medium viscosity also caused more calcite to form. Joshi et al. [136] studied the effects of a urease producing bacterium *Bacillus* sp. CT5 by adding the bacteria into cement mixture or spraying the bacteria on the concrete surface. Addition of bacteria into cement mixture delayed the setting time. Both methods led to the lowest sorptivity coefficient, water penetration, and chloride penetration. However, compressive strength of the addition method was higher. Analysis revealed that CaCO_3_ in the form of calcite and aragonite mostly precipitated in upper depth (0 mm–10 mm) of the samples but none in middle depth (20 mm–30 mm) and inner depth (40 mm–50 mm). Bacteria were found in middle depth for both methods, but were found in inner depth only for the addition method. Xu and Yao [137] added some non-ureolytic Bacillus genus together with calcium sources into concrete and studied the self-healing capability. Calcium glutamate was better than calcium lactate because calcium glutamate caused thicker transition zone which enhanced the bonding in the concrete. However, they also reported that healing agent was less effective than surface treatment due to different amount of activated bacteria and nutrient supply. Chu et al. [138] used a *Bacillus* sp. VS1 for biocementation of sand columns together with metal ions pretreatment. They found that intact bacteria suspension had better MICP ability than washed suspension of bacteria and supernatant. They also found that protease activity greatly reduced urease activity and therefore must be controlled. The study showed that surface coating of Ca^2+^, Fe^3+^, or Al^3+^ on sand enhanced bacteria cell adsorption by 31%. The author established some equations relating compressive strength and permeability to CaCO_3_ content to estimate time needed to achieve certain compressive strength or permeability. Li et al. [139] exposed a Bacillus genus to UV light and obtained a mutant strain LHUM107. Urea degradation efficiency of the strain greatly increased from 67% to 97% after mutation. The mutant showed potential to enhance the MICP process. Rizwan et al. [140] used two types of effective microorganism consortia containing yeast, lactic acid bacteria, and photosynthetic bacteria to make biocement. Setting time of cement paste increased with addition of the consortia solution. This method also required the addition of super plasticizer. The biocement had lower water adsorption than control sample. Highest compressive strength reported for the biocement was 89 MPa. Analysis revealed that mainly calcite and some wollastonite were formed by the effective microorganisms. Luo et al. [141] used some unknown spore forming alkali resistant bacteria to make self-healing concrete. The self-healing capability was 85% for crack width less than 0.3 mm, 50–70% for 0.3 mm–0.5 mm, and less than 30% for up to 0.8 mm within 20 days. Good healing was observed up to 28 days but then decreased greatly at 60 days to 90 days. They also reported that cracked concrete needed to be immersed in water to achieve good healing as self-healing at atmosphere with 90% relative humidity was quite low. In another study, the same group [142] reported crack healing of up to 0.48 mm wide cracks within 80 days and water permeability reduction up to 96% within 28 days of self-healing. Calcite was detected in all cases. Qian et al. [143] compared the performance of calcite and phosphate formed by bacteria on sheet glass interface. Various tests were conducted and the author concluded that bio-calcite was best among all samples in terms of intensity of interface interactions, strength per mass, and interfacial bonding strength. Mors and Jonkers [144] reported that the addition of a bacterial healing agent has insignificant effect on concrete strength but increased the self-healing capability to three times higher than normal concrete. They also proposed a method for its applications to reduce environmental impact and costs. Gat et al. [145] studied the stability of bacterial CaCO_3_ crystals in aqueous phase. CaCO_3_ dissolution was observed starting from 20 days after complete MICP and up to 30% CaCO_3_ loss was recorded at the end of experiment. This dissolution was caused by ammonia volatilization. Therefore, it was suggested to increase Ca^2+^ or CO_3_^2−^ to prevent ammonia volatilization or just remove ammonia after complete MICP. Ammonia volatilization effect was only observed on the surface or near the surface but not several cm into the soil. Liu et al. [146] studied the MICP of bacteria in activated sludge on aerobic granules of different sizes in the reactor for wastewater treatment. Local microenvironment varied due to different mass transfer resistance and thus affected the rate of MICP on granules with different sizes. More CaCO_3_ was formed on larger granules but very large granules (more than 700 µm) may limit MICP. Acetate metabolism enhanced MICP by increasing CO_3_^2−^ concentration and pH. Some bacteria consortia can sequester CO_2_ from atmosphere through their MICP process. The CO_2_ sequestration ability depends on the bacteria species in the consortia [147]. Wiktor and Jonkers [148] reported the application of MICP to heal cracks in a parking garage. Sodium silicate was added to the healing solution to provide rapid initial crack sealing (weaker sealing) and alkaline pH for MICP (stronger sealing). Mass loss due to freeze-thaw cycle was reduced from 3.6 kg/m^3^ to 1.9 kg/m^3^. All areas previously with heavy leaking only had a few localized dripping zones or no leaking after sealing. Jroundi et al. [149] used microorganisms obtained from historic gypsum plaster to treat gypsum plaster from 13–15th century. Analysis showed that 95% of the microorganisms were carbonatogenic and 10% produced acids with addition of glucose. The authors stated that bacteria can penetrate deeper into the sample compared to other conventional consolidants. Bacterial treated plaster was reported to have better drilling resistance, slightly decreased porosity, no significant color change, and 1.5–2% mass vaterite precipitated.

## 3. Remarks and Aspects for Future Studies

In recent years, *Sporosarcina pasteurii* is clearly the most studied bacterium for MICP followed by various Bacillus species including *Bacillus sphaericus*, *Bacillus megaterium*, *Bacillus subtilis*, and *Bacillus mucilaginous*. Other bacteria such as cyanobacteria, nitrate reducing bacteria, and sulfate reducing bacteria are also tested for their MICP potential. Researchers have also been diligently isolating bacteria from various environments in order to obtain new bacterial strains that can effectively cause MICP. The MICP experiments conducted are mostly biocementation of sand columns, consolidation of soil, development of self-healing mortar or concrete, and crack sealing. It is interesting to note that MICP processes have potential in heavy metal/ion removal from water samples [14,150,151,152].

Table 6 shows the performance of bacteria MICP in consolidation of sand and soil. Higher initial concentration of bacteria usually leads to better MICP performance because there are more cells available to induce CaCO_3_ precipitation. Higher concentrations of urea and CaCl_2_ also often lead to better MICP performance. Types of sand or soil greatly affect the strength of final MICP products. This may be due to different bacteria cells retention and penetration as well as distribution of CaCO_3_ crystals in the sand or soil. MICP seems to have lowest biocementation performance on poorly graded sands. Another factor that affects MICP performance is the method to introduce bacteria and biocementation solution into sand or soil to ensure uniform distribution across all volume. A lot of recent studies about consolidation of sand and soil used *Sporosarcina pasteurii* probably because it is the most established bacteria for MICP over the years. Potential of other types of bacteria should also be investigated.

Table 7 shows the performance of bacterial MICP in making bacterial concrete or mortar and their self-healing potential. A large variety of bacteria have been used to make bacterial concrete and mortar. Type of bacteria definitely affected performance of the final MICP products due to their differences in enzyme activity, size, and reaction pathway to precipitate CaCO_3_ crystals. Generally, these bacterial concretes or mortars have equal or better strength and durability compared to normal concretes or mortars. Bacterial concrete or mortar also had better self-healing capability, and 0.5 mm–1.0 mm wide cracks can be healed. Encapsulation of bacteria in carriers can enhance the MICP performance due to higher cell survivability. Addition of other substances such as fly ash or rice husk ash may improve or reduce MICP performance, thus they must be chosen wisely.

Many bacteria used to study MICP are ureolytic. They have high urease activity to catalyze the hydrolysis of urea and elevate surrounding pH, which leads to the formation of CaCO_3_. However, this process is sometimes criticized due to the formation of nitrogenous byproducts especially ammonium that can be harmful to living organisms and environment. These byproducts need to be converted into other harmless forms or completely removed after MICP is completed. Utilization of non-ureolytic bacteria can also solve this problem. Non-ureolytic bacteria consume other organic compounds such as lactate instead of urea to form carbonate ions. Some of them can even capture CO_2_ from the atmosphere and convert them into carbonate ions to form CaCO_3_ with calcium ions. This shows that MICP process can be developed to sequester CO_2_ and contribute in reducing greenhouse gas in the atmosphere. More studies can be conducted to explore this potential.

Harsh conditions such as high alkalinity and lack of nutrients greatly affect bacteria cell availability and MICP behavior. Genetic modification of bacteria may increase bacteria survivability and enzyme activity to enhance MICP process. In order to develop mutant bacteria, which are safe to use and good for MICP, integration of knowledge from different fields is required. Nevertheless, this can be a good aspect to be included in future studies. One of the limitations of current MICP technique is that CaCO_3_ crystals only precipitate on surface and in upper to middle parts of larger samples. MICP does not occur in deeper parts of the samples due to lack of necessary compounds there. Therefore, more studies can be conducted to ensure that CaCO_3_ crystals precipitate uniformly inside large samples. Application costs of the MICP technique can be reduced by using plant and animal waste materials as nutrients. Researchers from various places should explore the potential of local wastes to be used in MICP process. This can not only reduce costs but also increase sustainability of the process.

## 4. Conclusions

The MICP techniques show promising potential for applications in various fields such as construction, geotechnology, and nanotechnology. MICP can reduce OPC usage and enhance sustainability. MICP performances of various bacteria have been discussed in this paper. Some of the studies have indicated that the bacterial strains can extract carbon dioxide from air for the precipitation of CaCO_3_. On one hand, reduction of accumulation of greenhouse carbon dioxide is ensured and on the other hand cracks in the cement are sealed and healed. This technique is shown to be commonly used for biocementation of sand, consolidation of soil, and development of self-healing concrete. This technique can also apply for removal of heavy metals. Future studies are expected to further enhance the MICP performance, reduce its application costs, and increase its sustainability.

## Figures and Tables

**Table 1:** MICP with *Sporosarcina pasteurii* for sand and soil improvement.

Ingredients	Structure and Properties after MICP	Reference
Sand, clay	Increased tensile strength (40.8 kPa) and compressibility, decreased permeability (0.53 × 10^−7^ m/s)	[19]
Ottawa silica sand	Unconfined compressive strength (UCS) 1.3 MPa, flexure strength 0.95 MPa	[20]
Sand, metakaolin, OPC	OPC-MICP has best properties with UCS 1.2 MPa, water absorption 8%	[21]
Sand, PVA fiber	Highest UCS 1.6 MPa, highest splitting tensile strength 440 kPa, lowest permeability 1.05 × 10^−5^ m/s	[22]
Sand	Highest CS 3.29 MPa	[26]
Desert aeolian sand	Highest UCS 18 MPa, lowest permeability 0.92 × 10^−8^ m/s	[28]
Medium/fine sand	UCS 1.74 MPa, durability and water stability increased	[29]
Poorly graded course sand	UCS 525 kPa	[32]
Poorly graded sandy soil	UCS 400 kPa	[55]
Sandy soil	UCS 625 kPa, permeability 1.8 × 10^−7^ m/s	[56]
Sandy soil	Highest 6.4 MPa after 4 treatments, permeability 1.0 × 10^−5^ m/s	[57]

**Table 2:** Construction materials made by MICP with *Sporosarcina pasteurii.*

Materials	Structure and Properties after MICP	Reference
Bio-brick from silica rich masonry sand	Highest CS 2.2 MPa	[37]
Red brick (treatment)	CS 7.54 MPa, reduce water absorption by 49% after treatment	[39]
Concrete with light weight aggregates	Highest CS 40 MPa, lowest water absorption 5%	[58]
Bio-mortar	Highest CS 39.6 MPa, tensile strength 37% higher than normal mortar	[59]
Bio-mortar	Highest UCS 43 MPa, lowest water absorption 2.5%	[42]
Bio-mortar	Highest UCS 44 MPa	[60]
Bio-mortar	Highest CS 54/70 MPa at 7/28 days curing	[43]
Bio-mortar with superplasticizers	Crack width healed 0.35 mm	[44]
Bio-cement	CS 42 MPa, water absorption 21%	[45]
Bio-mortar	Crack width healed 0.41 mm, water adsorption restored 95%, CS restored 84%	[61]
Bio-mortar	Crack width healed 0.27 mm, CS restored 63%	[62]
Bio-mortar with fiber and zeolite as bacteria carriers	CS 70/100 MPa at 7/270 days	[63]
Geopolymer	Self-healing observed in 1 month old sample	[64]

**Table 3:** Materials produced using MICP with *Bacillus sphaericus.*

Material	Structure Properties after MICP	Reference
Bio-mortar with biochar, superabsorbent polymer, polypropylene fiber	CS 35–60 MPa, flexure strength 9–12 MPa Crack width healed up to 0.9 mm, water penetration restored 70%	[68]
Bio-concrete with fly ash	Highest CS 32.5 MPa, highest tensile strength 4.1 MPa, highest flexure strength 3.5 MPa	[73]
Bio-concrete with fly ash	CS 30–40 MPa, tensile strength 2.9–5.0 MPa	[74]
Bonding repair mortar	Highest slant shear strength 17 MPa	[75]
Industrial ceramic aggregates (treatment)	Water absorption 6–16%, weight gained 3–7%	[76]

**Table 4:** Materials produced using MICP with *Bacillus megaterium.*

Material	Structure and Properties after MICP	Reference
Treat sand column with varying grain size	Up to 30% CaCO_3_ formation	[82]
Bio-concrete with recycled aggregates, nanosilica	Water absorption 5%, void volume 10%	[83]
Bio-mortar	Highest CS 36 MPa, permeability 5 × 10^−5^ m/s	[84]

**Table 5:** Materials produced using MICP with *Bacillus subtilis.*

Material	Structure and Properties after MICP	Reference
Bio-mortar	Highest CS 50 MPa, lowest water absorption 5% Crack width healed up to 1.2 mm	[89]
Bio-concrete	Highest CS 44 MPa Self-healing observed	[90]
Bio-shotcrete	Highest CS 34 MPa, highest tensile strength 3.4 MPa, lowest water absorption 6.2% Self-healing observed	[92]
Sand column (mixture of *B. subtilis* and *S. pasteurii*)	UCS 1.69 MPa, permeability 1.06 × 10^−5^ m/s	[93]

**Table 6:** Biocementation of sand and soil through MICP.

Bacteria (Initial Concentration)	Sand/Soil	Cementation Solution	Performance	Reference
*Sporosarcina pasteurii* + *Bacillus subtilis* (OD_600_ = 1.2)	Sandy soil	2 M urea + 1 M CaCl_2_	UCS = 1.69 MPa Permeability = 1.06 × 10^−5^ m/s	[93]
*Sporosarcina pasteurii* (OD_600_ = 0.6)	Ottawa silica sand	0.5 M (urea + CaCl_2_)	UCS = 1.30 MPa	[20]
*Sporosarcina pasteurii* (OD_600_ = 1.0)	Commercial sand + white kaolin clay	0.5 M (urea + CaCl_2_)	Tensile strength = 0.04 MPa Permeability = 0.53 × 10^−7^ m/s	[19]
*Sporosarcina pasteurii* (OD_600_ = 1.9–2.4)	Desert aeolian sand	2.5 M (urea + CaCl_2_)	UCS = 18 MPa Permeability = 0.92 × 10^−7^ m/s	[28]
*Sporosarcina pasteurii* (OD_600_ = 2.3)	Natural SiO_2_ sand	1.0 M (urea + CaCl_2_)	UCS 1.74 MPa	[29]
*Sporosarcina pasteurii* (OD_600_ = 0.6)	Ottawa silica sand	0.75 M (urea + CaCl_2_)	UCS = 6.4 MPa Permeability = 1.00 × 10^−5^ m/s	[40]
*Sporosarcina pasteurii* (Not provided)	Poorly graded medium sand	1.0 M (urea + CaCl_2_)	Surface strength = 4.83 MPa	[27]
*Sporosarcina pasteurii* (OD_600_ = 2.0)	Loose sand	0.5 M (urea + CaCl_2_)	UCS (MICP) = 0.10 MPa UCS (MICP + OPC) = 1.10 MPa Water adsorption (MICP) = 11% Water adsorption (MICP + OPC) = 8%	[21]
*Sporosarcina pasteurii* (OD_600_ = 3.5)	Standard sand	0.5 M (urea + CaCl_2_)	UCS = 3.29 MPa	[26]
*Sporosarcina pasteurii* (OD_600_ = 1.5)	Sandy soil	3 mM urea + 2 mM CaCl_2_	UCS = 0.63 MPa Permeability = 1.80 × 10^−5^ m/s	[39]
*Sporosarcina pasteurii* (1.5 g/L)	Ottawa silica sand + PVA fiber	0.5 M (urea + CaCl_2_)	UCS = 2.20 MPa Splitting tensile strength = 0.60 MPa Permeability = 4.00 × 10^−7^ m/s	[22]
*Sporosarcina pasteurii* (OD_600_ = 2.5)	Poorly graded SiO_2_ sand	1.0 M (urea + CaCl_2_)	UCS = 0.53 MPa	[32]
*Sporosarcina pasteurii* (OD_600_ > 2)	Poorly graded sandy silica	1.0 M (urea + CaCl_2_)	UCS = 0.50 MPa Permeability = 0.85 × 10^−6^ m/s	[31]

**Table 7:** Performance of MICP by various bacteria in making concrete and mortar.

Bacteria (Initial Concentration)	Other Additives	Performance	Reference
*Bacillus sphaericus* (10^10^ cell/mL)	Biochar, PP fiber, SAP	Compressive strength = 53.0 MPa Water penetration = 9.0 mm Crack width healed = 0.9 mm	[68]
*Bacillus sphaericus* (Not provided)	Fly ash	Compressive strength = 32.5 MPa	[73]
*Bacillus* sp. CT5 (OD_600_ = 0.5)	-	Compressive strength = 46.0 MPa Water penetration = 14.2 mm	[135]
*Bacillus subtilis* (10^3^–10^7^ cell/mL)	-	Compressive strength = 54.0 MPa Water adsorption = 4% Crack width healed = 1.2 mm	[89]
*Lysinibacillus* sp. I13 (Not provided)	Fly ash	Compressive strength = 33.6 MPa *Able to heal cracks but no exact values provided	[123]
*Sporosarcina pasteurii* (10^9^ cell/mL)	Calcium sulpho-aluminate cement, silica fume	Compressive strength = 46.8 MPa Crack width healed = 0.4 mm	[55]
*Sporosarcina pasteurii* (8 × 10^8^ cfu/mL)	-	Compressive strength = 70.0 MPa	[50]
*Sporosarcina pasteurii* (10^6^ cell/mL)	Zeolite, fiber reinforced	Compressive strength = 84.0 MPa Water penetration = 1.5 mm Crack width healed = 0.1 mm	[57]
*Sporosarcina pasteurii* (10^8^–10^9^ cell/mL)	-	Compressive strength = 39.6 MPa	[47]
*Bacillus cohnii* (5.2 × 10^8^ cell/mL)	Expanded pearlite	Crack width healed = 0.8 mm	[108]
*Bacillus sphaericus* (10^5^ cell/mL)	Fly ash	Compressive strength = 40.4 MPa	[72]
*Bacillus cereus* (5 × 10^8^ cfu/mL)	Metakaolin	Compressive strength = 40.2 MPa	[105]
*Bacillus aerius* (10^5^ cell/mL)	Cement baghouse filter dust	Compressive strength = 36.3 MPa Water adsorption = 1.2%	[125]
*Bacillus aerius* (10^5^ cell/mL)	Rice husk ash	Compressive strength = 35.0 MPa Water adsorption = 1.1%	[126]
Bacillus mucilaginous (10^8^–10^9^ cell/mL)	Ceramsite	Crack width healed = 0.5 mm Water permeability = 0.8 × 10^−7^ m/s	[96]
*Bacillus megaterium* (OD_600_ = 1.5)	-	Compressive strength = 35.0 MPa	[81]
*Pseudomonas aeruginosa* *Diaphorobacter nitroreducens* (Not provided)	Granular activated carbon	Crack width healed = 0.5 mm	[113]
Soil bacteria (OD_600_ = 0.866)	-	Compressive strength = 48.0 MPa Water adsorption = 5.8%	[132]
